# Caregiver Burden in Patients Receiving Ranibizumab Therapy for Neovascular Age Related Macular Degeneration

**DOI:** 10.1371/journal.pone.0129361

**Published:** 2015-06-09

**Authors:** Rishma Gohil, Roxanne Crosby-Nwaobi, Angus Forbes, Ben Burton, Phil Hykin, Sobha Sivaprasad

**Affiliations:** 1 NIHR Moorfields Biomedical Research Centre, London, United Kingdom; 2 King’s College London, London, United Kingdom; 3 James Paget University Hospital, Great Yarmouth, United Kingdom; 4 King’s College Hospital, London, United Kingdom; Sun Yat-sen University, CHINA

## Abstract

**Purpose:**

To assess the caregiver burden and factors determining the burden in patients receiving ranibizumab therapy for neovascular AMD (nAMD).

**Methods:**

This is a cross-sectional questionnaire survey of 250 matched patient caregiver dyads across three large ophthalmic treatment centres in United Kingdom. The primary outcome was the subjective caregiver burden measured using caregiver reaction assessment scale (CRA). Objective caregiver burden was determined by the caregiver tasks and level of care provided. The factors that may predict the caregiver burden such as the patient’s visual acuity of the better eye and vision related quality of life, demographics, satisfaction and support provided by the healthcare and the health status of the dyads were also collected and assessed in a hierarchical regression model.

**Results:**

The mean CRA score was 3.2±0.5, similar to the score reported by caregivers for atrial fibrillation who require regular hospital appointments for monitoring their thromboprophylaxis. Caregiver tasks including accompanying for hospital appointments for eye treatment and patient’s visual acuity in the better eye were the biggest contributors to the caregiver burden hierarchical model explaining 18% and 11% of the variance respectively.

**Conclusion:**

Ranibizumab therapy for nAMD is associated with significant caregiver burden. Both disease impact and treatment frequency contributed to the overall burden.

## Introduction

Advanced age related macular degeneration (AMD) is a common cause of visual impairment in the older population.[1] The 15-year cumulative incidence of advanced AMD in individuals 75 years of age or older is 8%.[2] The wet form of AMD constitutes 10–15% of cases and is caused by neovascularisation (nAMD) under the retina and the loss of vision can be dramatic over a few weeks. If left untreated, nAMD results in a scar in the central retina with severe visual loss. Visual morbidity caused by nAMD is a significant public health problem. In the United Kingdom (UK), 192,000 people aged 75 years or older were reported to be visually impaired due to advanced AMD in 2004 before the availability of treatment options that could improve visual acuity.[3] Visual impairment in the older age group is associated with social and functional decline, the need to access community support services, depression, falls, nursing home placement and increased mortality.[4–7] A study on care utilization of patients with AMD in the UK indicated that visual impairment is not the only factor why patients require care. Care utilization was predicted by age, visual acuity in the better eye and living arrangement. This study was also done before the advent of current treatment options for nAMD.[8] The caregiver burden in the current era when treatment options are available to improve visual acuity in patients with this condition is unknown.

New treatment options with repeated intravitreal injections of inhibitors of vascular endothelial growth factor (VEGF) for nAMD have had a significant positive impact on the prevalence of blindness due to this condition.[9] The burden of this age-related disorder is now more related to visual morbidity due to poor quality of vision rather than blindness.[10]

The first intravitreal anti-VEGF licensed for this condition is ranibizumab. Currently, monthly monitoring and pro-re-nata (PRN) dosing of intravitreal ranibizumab is the recommended approach in the National Health Service in the UK since 2008. However, monthly monitoring is often difficult to accommodate in the retinal clinics. Therefore, a 4–6 weekly monitoring is usually adopted with the aim of providing optimal outcomes within the constraints of limited clinic capacity. The impact of this intense treatment regimen on caregiver burden is also unknown. In particular, approximately 30% of elderly patients do not drive[11] and these patients are also constrained because their pupils are dilated at every visit to the eye clinic. Their advancing age and associated physical co-morbidities further preclude travelling independently. It is therefore important to quantify the current caregiver burden of nAMD in terms of both disease impact on visual acuity and treatment frequency to help us understand the impact of this condition on carers compared to other chronic diseases and conditions that require frequent hospital appointments.

The aim of this study was to estimate the subjective and objective caregiver burden for patients with nAMD receiving PRN ranibizumab therapy and to determine the factors that influence the burden.

### Ethics

The study was approved by the National Research Ethics Service Committee (13/WA/0032) and conducted according to the tenets of the Declaration of Helsinki. Written informed consent was obtained from all participants prior to completion of the questionnaire.

## Methods

### Patient and Caregiver Recruitment

This cross-sectional questionnaire-based survey was conducted on 250 patient-caregiver pairs from 3 public ophthalmic treatment centres in the United Kingdom (North London, South London and East Anglia). The pairs were recruited from a convenience sample of patients utilising these three services for treatment of nAMD. The protocol for treatment of nAMD is similar in all 3 centres with clinical audits from each centre indicating similar treatment outcomes. All patients were initiated on a loading phase of monthly ranibizumab therapy for 3 months followed by 4–6 weekly review and PRN dosing. In a routine clinic appointment, the patients undergo visual acuity tests, a macular scan using optical coherence tomography (OCT), slit-lamp biomicroscopy and then injected with ranibizumab if deemed necessary. The clinic visit time may range from 1–4 hours depending on the waiting time for evaluation and treatment.

Inclusion criteria for caregivers were that they were aged > 18 years and identified themselves as the primary caregiver. A primary caregiver was defined as “any person who, without being a professional or belonging to a social support network, and in some way, is directly implicated in the patient’s eye care or is directly affected by the patient’s health problem”.[12] Individuals who received financial compensation for their services and those who did not speak fluent English were excluded. Patient participants were included if they had ≥ 6 months of follow-up after initiation of ranibizumab therapy, this ensured that all caregivers had been supporting the patients following a minimum treatment exposure. Patients were excluded if their associated medical condition rendered them incapable of making an informed decision to give consent. Patients without accompanying caregivers were excluded from this questionnaire survey. The proportions of patients with caregivers in each clinic and the pairs approached were ascertained to assess the response rate.

### Measures for caregivers

#### Subjective caregiver burden assessment

The subjective caregiver burden refers to how the caregiver perceives the impact of the objective burden of caregiving. was evaluated using the validated Caregiver Reaction Assessment (CRA).[13] The CRA has been used extensively with family caregiver populations including elderly population with good internal consistency and content and construct validity testing.[14, 15] The instrument is a simple self-rated burden scale consisting of 24 items representing 5 dimensions of the caregiving situation. The five dimensions of caregiver reactions include the impact of caregiving on the caregiver's schedule, impact of caregiving on caregiver's financial situation, degree of family support, impact of caregiving on caregiver's health status, and the degree to which the caregiver views on self-esteem. Each item is answered using a 5-point Likert scale with responses ranging from 1 (strongly agree) to 5 (strongly disagree). The composite scores are computed as averages of the items within each dimension, ranging from 1.0 to 5.0. Higher scores on the negative dimensions represent higher levels of perceived burden (the exception to this is the self- esteem scale, however for the purpose of our analysis we inverted this scale for consistency of interpretation). Therefore, the higher the score, the higher is the perceived burden. We tested the Cronbach α values for each scale and these ranged from 0.62 and 0.83 for the separate subscales.

### Objective caregiver burden assessment

The objective burden of a caregiver refers to the caregiver tasks and the level of care provided for the patient.[16] We gathered data on general caregiver activities and eye-related activities. For the general caregiver activities, we asked the caregivers whether they performed any of the pre-specified basic and instrumental daily living activities in the last year using a validated questionnaire.[17] Basic self-care activities included toileting; feeding; dressing; grooming; physical ambulation; and bathing. The instrumental activities were telephone usage; shopping; food preparation; housekeeping; doing laundry; mode of transportation; responsibility for patient’s medications; and handling finances. Composite scales were derived from a factor analysis (principal components and varimox rotation) of items (n+19) on these caring activities. Five-point increments from no help to a lot of help were used in scoring, with a maximum score of 100. For the purposes of statistical analysis, we re-defined the amount of assistance required into low (no help or a little help given), medium (a moderate amount of help given) or high (quite a lot or a lot of help given).

For eye-related activities, we designed questions to quantify the impact of the ranibizumab therapy: the number of appointments attended; the average time taken for appointments; time taken away from work; loss of income; and whether they administered eye drops to the patient post-injection. The responses for these items were reported as means or proportion.

### Health status assessment

We used both the EuroQoL instrument and pre-specified questions on specific eye care related healthcare problems to measure the health-related quality of life of caregivers.

The EuroQoL instrument is validated and has a descriptive part (EQ-5D-3L) and a visual analogue score (EQ-VAS).[18] The EQ-5D-3L measures the current health status on five domains (mobility; self-care; usual activity; pain/discomfort; anxiety/depression) with three levels of severity per domain as no problems, some problems or severe problems. EQ-5D-3L also generates a composite index value based on the 5 domains to indicate overall health. The self-reported health state is then valued using a visual analogue scale with a range of 0 (worst imaginable health state) to 100 (best imaginable health state). Both the patient and caregiver completed the EQ-5D-3L and we estimated a variable for dyadic health comprising of the mean of the patient and caregiver EQ-5D index score. This dyadic value estimated the collective health of each patient /caregiver pair.

In addition, we also assessed the patient and caregiver perception of the degree of visual disability so that we could better understand the impact of this perception on overall health of the caregivers. We also requested caregivers to identify whether caring for their patient requiring treatment for nAMD created any specific health problems. These health problems were feeling tired, a bad back, feeling anxious or stressed, sleepless nights; feeling sad or depressed; shortness of breath; and relationship problems as a result of caring for the patient’s nAMD. These problems were rated as ‘no or minimal’, ‘moderate’ or ‘severe’.

### Predictors of caregiver burden

We also assessed other factors that may influence the subjective caregiver burden.

1. *Demographic data* collected on the carer and patient included age, gender, education level, occupation, co-resident with patient and relationship to the patient.
2. *Healthcare support*: Questions were designed to establish the support provided to the accompanying caregivers to cope with their caregiver burden. These questions to the caregivers addressed whether their concerns were attended to and they received reassurance about the potential visual impairment of their patient. Information on their caring role and whether their needs were assessed and supported were also collected. These questions rated the level of support as ‘not at all’, ‘some’ and ‘often’.
3. Healthcare satisfaction was also assessed with Care Satisfaction Questionnaire-8 (CSQ-8).[19] This validated tool indicates the level of satisfaction of the healthcare provided by scoring from 8 (no satisfaction) to 32 (maximum satisfaction).
4. *Patient disease impact*: Data on patient disease were collected to assess the contribution to caregiver burden. The patient data included demographic details, visual acuity in better eye, number of ranibizumab injections to date, number of clinic appointments and total follow-up period. Self-reported vision-related health status was measured using the National Eye Institute Visual Function Questionnaire (NEI VFQ-25).[20] The NEI VFQ-25 is 25-item questionnaire used to generate a single composite score that ranges from 0 to 100, with 100 being the maximum visual function; The overall composite score is the mean of the subscale scores for general vision; ocular pain; near activities; distance activities; driving; colour vision; peripheral vision; and vision-specific social functioning, mental health, role difficulties, and dependency on others to perform visual tasks.

### Statistical analysis

**Fig 1:**
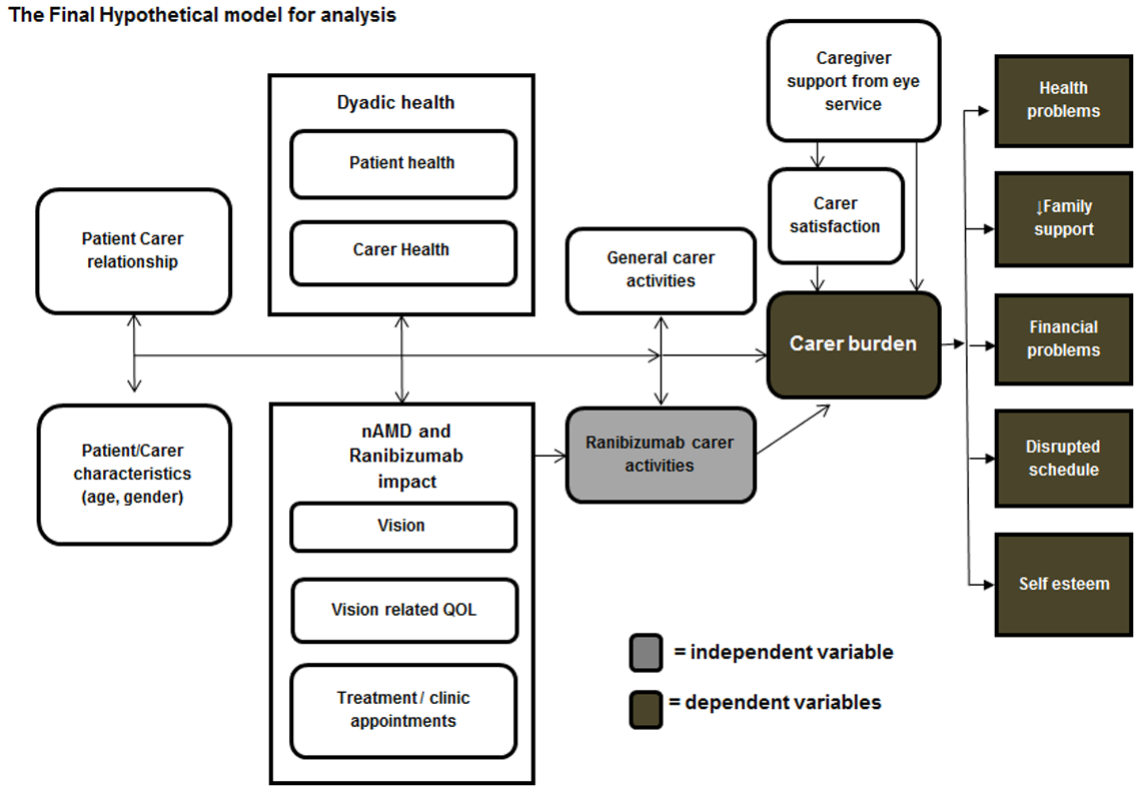
The final hierarchical model for analysis. The final hierarchical model shows the impact of caregiver activities on the CRA subscales.

The primary outcome was the subjective caregiver burden (mean CRA score). We created a hierarchical model in order to test the hypothesis that the carer activities related to eye therapy are independently associated with the CRA score (Fig 1). Descriptive statistics were compiled to provide the demographic characteristics of caregivers; clinical and demographic details of the patients; objective caregiver burden; healthcare support; healthcare satisfaction and quality of life of caregivers and patients. Correlations between these covariates of patient and caregiver characteristics and the CRA composite and each of the five CRA domain score were computed to assess candidacy for inclusion into the model. Covariates with a p-value < 0.2 were included into the model.

All the statistical analyses were performed using IBM SPSS for Windows, version 20.014. An alpha of 0.05 defined statistical significance.

## Results

A total of 273 patients-caregiver dyads were approached to recruit 250 pairs (91.5% response rate). On an average, 72% of the patients in the clinic was accompanied by informal caregivers. The characteristic of the caregivers and patients are summarised in Table 1.

**Table 1:** Baseline characteristics of caregivers and patients.

		*N (%) or Mean± SD*	
*Characteristics*		Caregivers (n = 250)	Patients (n = 250)
Age in years: mean ±SD	All patients	64.4±13.5	79.6±8.8
	≥80 years	37(15.0)	138(55.2)
	70–80	55(22.4)	81(32.4)
	60–69	71(28.9)	24(9.6)
	50–59	53(21.5)	7(2.8)
Gender	Male	93(37.2)	64(25.6)
	Female	157(62.8)	186(74.4)
Marital status	Single	29(11.6)	11(4.4)
	Married or Partner	191(76.4)	134(53.6)
	Separated or Divorced	17(6.8)	19(7.6)
	Widowed	12(4.8)	86(34.4)
Employment status	Employed	87(34.8)	14(5.6)
	On sick leave	8(3.2)	0(0)
	Unemployed	15(6.0)	1(0.4)
	Retired	136(54.4)	235(94.0)
Relationship to patient	Spouse	97(38.8)	NA
	Family member	115(46.0)	NA
	Friend	35(14.0)	NA
Live with patient	Yes	115(46.4)	NA
	No	133(53.6)	NA
Eye & treatment characteristics	EQ-5D index	0.80±0.21	0.68±0.22
	EQ-5D VAS	74.5±18.2	64.6±15.0
	Dyadic value of EQ-5D index	0.74±0.16	

EQ-5D = EuroQol 5D health index; EQ-5D VAS = EuroQol 5D visual analogue scale

### Subjective caregiver burden

Table 2 shows the mean scores of the respondents on the total score as well as the scores on the 5 CRA dimensions. There were statistically significant differences in the total perceived caregiver burden and the subscales of the CRA depending on the caregiver’s relationship to the patient with the exception of the self-esteem subscale. In general, caregivers who were either siblings or offspring reported a higher caregiver burden than friends or spouses. This may be explained by the fact that the siblings and off-spring were younger (mean age 56 years compared to 66 and 74 years for friends and spouses respectively) and more likely to be employed, with greater potential disruption to their daily routine. When stratified by age of patients, the total CRA score was significantly higher for cares of older patients (aged ≥66 years) and this was driven by the impact on finances.

**Table 2:** Subjective caregiver burden CRA (mean ± SD): 1) with reference to relationship of caregiver to patient, 2) stratified by age of carer.

CRA	Overall score	CRA score for each caregiver group			P value	Overall score	CRA score for each age group		P value
		Spouse (n = 97)	Family (n = 115)	Friend (n = 35)			≤65 (n = 128)	≥66 (n = 118)	
Self-esteem	2.0±0.44	2.0±0.39	2.1±0.42	2.0±0.44	0.208	2.0±0.44	2.0±0.44	2.0±0.44	0.931
Impact of finances	3.9±0.88	4.0±0.88	3.7±0.85	3.9±0.88	**0.005**	3.9±0.88	3.7±0.87	4.1±0.85	**0.002**
Impact on health	2.9±0.68	2.9±0.70	2.8±0.60	2.9±0.68	**<0.001**	2.9±0.68	2.9±0.59	3.0±0.77	0.09
Disrupted schedule	3.3±1.14	3.3±1.21	3.2±1.1	3.3±1.14	**0.023**	3.3±1.14	3.3±1.13	3.4±1.17	0.522
Lack of family support	3.8±0.85	4.0±0.82	3.7±0.89	3.8±0.85	**0.015**	3.8±0.85	3.7±0.82	3.9±0.87	0.139
CRA total	3.2±0.51	3.3±0.49	3.1±0.51	3.2±0.51	**0.011**	3.2±0.51	3.1±0.50	3.3±0.50	**0.006**

Where CRA = Caregiver’s Reaction Assessment scale

### Objective caregiver burden

The proportion of caregivers providing support for patients to perform basic and instrumental daily living activities are shown in Table 3. 12% required help from caregivers for basic self-care while 47% required caregiver to aid them with instrumental daily activities. In terms of eye care support, 74% of the patients required support from caregivers. The most common eye-related care activity undertaken was accompanying patients for their eye clinic appointments, with 70.8% carers having attended ≥10 appointments over the last year. On an average, the caregivers reported that 70% spent at least half a day and 43.6% spent most of the day assisting their patient with their clinic visit every 4–6 weeks. 24.8% of caregivers required time off work to support patients with their eye treatment, and 10% of them reported that this led to a loss of income.

**Table 3:** Carer role in eye care and general support.

*Eye care support (in the last year)*				
Number of appointments:	**≤3**	**4–6**	**7–9**	**≥10**
	23(9.2)	37(14.8)	13(5.2)	177(70.8)
Time spent on appointment:				
≥half of day	175(70.0)			
most of day	109(43.6)			
Taken time off work:	62(24.8)			
Loss of income during visits:	29(11.6)			
Administer eye drops	63(25.2)			
*Carer activity domains (score 0–100)*				
	Low n(%)	Medium n(%)	High n(%)	Mean (SD)
Eye care support	64(26)	96(39)	89(35)	51 (±31)
Instrumental ADL	132(53)	73(29)	39(18)	35 (±27)
Basic ADL	216(88)	23(9)	7(3)	12 (±18)

Where ADL = Activities of Daily Living

### Health status

Overall, as expected, caregivers scored higher on EQ-5D than the patients (age-adjusted) with anxiety/depression being particularly prevalent in the patient group (68%). The dyadic health status, which describes the combined health of the patients and caregivers showed a high level of shared health (mean = 0.74 ±0.16), with a range from 0.27 to 1.00. The carer’s perception of patient’s symptoms of nAMD and visual difficulties experienced by the patient also contributed to the carer’s health status. 52 (21.0%) of carers rated the patient’s symptoms as mild, 145 (58.5%) as moderate and 51 (20.6%) as severe.

This rating of perceived symptoms of nAMD correlated with carers’ perception of the visual difficulty experienced by patients. 45 (18.3%) rated their patient’s visual difficulty as mild, 151 (61.4%) as moderate and 50 (20.3%) as severe. When asked about specific caregiver health problems related to the patient’s symptoms and visual difficulties, anxiety (38.8%), tiredness (39.6%) and depression (29.6%) were the most commonly experienced problems.

### Healthcare support and care satisfaction perceived by caregivers

The level and type of support received from the health service and the caregiver’s level of satisfaction with the care received are presented in Table 4. The responses highlighted that most carers receive or access little or no support from the healthcare support available. In terms of care satisfaction, caregivers reported a much higher level of satisfaction than did the patients.

**Table 4:** Healthcare support and Healthcare satisfaction reported by caregivers.

***Level of health service support***	Not all	Some	Often
Responding to worries/concerns	117(46.8)	48(19.2)	85(34.0)
Reassurance about blindness	149(59.6)	55(22.0)	46(18.4)
Information about nAMD	123(49.2)	40(16.0)	87(34.8)
Help to understand nAMD	123(49.2)	38(15.2)	89(35.6)
Information on caring role	171(68.4)	30(12.0)	49(19.6)
Arrangement of other services	202(80.8)	29(11.6)	19(7.6)
Assessment of caregiver needs	117(46.8)	31(12.4)	102(40.8)
Caregiver support given	205(82.0)	26(10.4)	20(7.6)
Practical advice given	206(82.4)	24(9.6)	20(8.0)
***Healthcare satisfaction***	Caregiver	Patient	
CSQ-8	28.4±4.1	15.7±1.8	

Where nAMD = neovascular Age-related Macular Degeneration; CSQ-8 = Care Satisfaction Questionnaire-8.

### Disease impact

The mean age of the patients was 79.6±8.8 years. The mean duration of treatment for nAMD was 36.2±20.7 months. The mean number of injections given was 12.3±9.7. The mean visual acuity letter score (ETDRS letters) and the NEI-VFQ 25 scores were 67.6±17.2 ETDRS letters and 54.7±23.0 respectively. These parameters are further stratified in Table 5 to illustrate the disease impact. Ninety-seven (38.8%) were undergoing ranibizumab injections in both eyes and 153 patients were receiving ranibizumab injections in only one eye. The mean visual acuity in the better eye and worse eye in this group with unilateral treatment were 69.2±18.3 and 37.1±31.1 ETDRS letters respectively.

**Table 5:** The clinical description of the patients included in this study.

	N = 250
*Visual acuity in better eye*	
≥74 ETDRS letters	122(48.8)
54–73 ETDRS letters	93(37.2)
37–53 ETDRS letters	20(8.0)
<37 letters	15(6.0)
*Visual acuity in worse eye*	
≥74 ETDRS letters	27(10.8)
54–73 ETDRS letters	69(27.6)
37–53 ETDRS letters	31(12.4)
<37 letters	123(49.2)
*NEI-VFQ 25 total composite score*	
≥90	11(4.8)
80–89	30(13.1)
70–79	33(14.4)
60–69	22(9.6)
50–59	29(12.7)
40–49	30(13.1)
30–39	32(14.0)
<30	42(18.3)
*Number of appointments to date*	
≤ 3	20(8.1)
4–6	23(9.3)
7–9	33(13.4)
≥ 10	132(53.4)
*Number of ranibizumab injections*	
≤ 3	37(15.0)
4–6	45(18.2)
7–9	33(13.4)
≥ 10	132(53.4)

### The relationship between subjective and objective caregiver burden

Bivariate analysis showed that increased frequency of the caregiver activity (objective burden) resulted in increased caregiver burden on the CRA scales (subjective burden). The final hierarchical regression model (Table 6) explained 32% of the variance in the CRA scores (F = 9.3, df = 1, p = 0.003). Objective burden in the form of catering to patient’s daily living activities and eye treatment related activities were the biggest contributors to the model explaining 18% and 11% of the variance respectively. The relationships were positively associated indicating that more activity increased burden. Importantly the eye care activity contributed most in the caregiving block within the analysis, indicating that supporting a patient on ranibizumab is associated with increased subjective caregiver burden. Satisfaction with care was significant in the model with a modest effect. Patient—caregiver dyadic health was not significant in the model. Modelling of the impact of caregiver activities on the CRA subscales showed that eye care related activity (p = 0.009) was positively associated with financial burden. Daily living activity (p = 0.020) was inversely associated indicating that the additional input of eye care caused increased caregiver burden rather than mere assistance with daily living which may be confounded by other co-morbidities. Eye care activity also impacted negatively on the level of family support received (p = 0.032) and the caregiver’s self-esteem (p = 0.042). The combined health of the patient and caregiver (index dyad) was positively associated with a disruption in schedule. Patients who reported a high quality of life with respect to nAMD were more likely to have better family support (p = 0.018).

**Table 6:** Hierarchical regression models predicting caregiver burden (CRA) in patients with nAMD.

Models	Variable statistics full model		Model statistics					
	Stand. β	Sig.	Block	Δ*R* ^2^	Adj. *R* ^2^	*R* ^2^ change	*F*-change	*P*-value
Model: Subjective Caregiver Burden (CRA)								
**Demographics:**								
Caregiver female gender	-0.171	0.013	1	0.029	0.024	0.029	6.273	**0.013**
**Patient disease impact:**								
Vision related QoL	-0.153	<0.001	2					
Vision in the better eye	-0.005	0.946	2					
Number of injections	-0.039	0.553	2	0.135	0.118	0.106	8.424	**<0.001**
**Objective caregiver burden**								
Instrumental daily living activities	-0.206	0.010	3					
Basic daily living activities	-0.193	0.004	3					
Eye care activities	-0.162	0.029	3	0.313	0.289	0.177	17.457	**<0.001**
**Other caregiver variables**								
Caregiver healthcare support								
Healthcare satisfaction	0.182	0.003	4	0.343	0.317	0.030	9.307	**0.003**
**Health status**								
EQ-5D dyad score	0.099	0.098	5	0.352	0.323	0.009	2.757	0.098

Where CRA = Caregiver’s Reaction Assessment scale; nAMD = neovascular Age-related Macular Degeneration; QoL = quality

## Discussion

This study demonstrates that caregiver burden is significant in patients with nAMD receiving ranibizumab therapy. While in part this burden may be explained by the advanced age of the patients and contribution of nAMD-related visual impairment, the modelling shows that activities related to the treatment of the condition also has significant impact on carer utilization amongst these individuals. These activities included taking patients to hospital appointments, organising medical appointments and supporting patients with their medication.

We noted that 72% of patients who attend the intravitreal injection clinics are accompanied by informal cares concurring with a previous report that indicated that majority of AMD patients rely on informal care from their families or friends rather than formal care. We have excluded patients requiring formal care in this study. We would expect the caregiver burden to be even more significant if formal care is also considered as previous studies have reported that patients with visual impairment are more prone to require community or institutional support.

The patient group in this study is generalizable to the nAMD cohort seen in intravitreal injection clinics based on the mean age of the patients, their visual acuity scores and average number of injections over the mean length of follow-up observed in other reports on real-life experience of using ranibizumab therapy for this condition. However, the patients in this study were treated with ranibizumab on a PRN regimen only so we will not be able to translate our results to other anti-VEGF agents or to other treatment regimens. Despite these factors, the study provides an accurate estimation of caregiver burden in nAMD patients undergoing frequent follow-ups and treatment in a hospital environment.

Significant emphasis has been placed on visual impairment driven caregiver burden in this condition. However, approximately 50% of the patients had visual acuity of 74 letters or better (Snellen 6/12 or better) in the better seeing eye suggesting that either visual acuity score is not an accurate measure of visual morbidity or that visual acuity is not the most important contributor of the caregiver burden. The mean vision related quality of life for this cohort of patients was 54.7±23.0, which is lower than the measurements obtained in clinical trial settings indicating the boarder mix of patients receiving treatment in real life. The mean age of the patients was 79.6 years with 55.2% being 80 years or older. Therefore, age and associated co-morbidities are added contributory factors. The EQ-5D is designed to measure decrements in health and does not map accurately to NEI VFQ-25.[21] Therefore, using both questionnaires together have indeed aided in assessing the whole contribution of health states of the patient group to the caregiver burden. The mean scores of the EQ 5D in this patient group was 0.68 and is comparable to the age-matched scores of this questionnaire obtained in a national survey of individuals in the UK.

In terms of the level of burden experienced by these caregivers, it is possible to compare the level of this burden with other disease populations. These comparisons suggest that nAMD caregivers experience a level of caregiver burden equivalent to caregivers for rheumatoid arthritis and multiple sclerosis[22, 23] and higher than patients with colorectal cancer.[24] The level of burden observed in our study is also similar to the scores on the CRA burden scales obtained from caregivers of patients with atrial fibrillation who require regular hospital appointments for monitoring their thromboprophylaxis.[25]

In addition to elevated caregiver burden, this study shows that the level of support caregivers receive is limited. Over half of the respondents reported that that they had not been given information about nAMD and its treatment, potential loss of vision or any support that might be available for them in their caregiver role. Therefore, it would seem to be important to improve the information and supportive resources for caregivers of patients with nAMD. This study has also shown that there may be variations in the needs of different groups of caregivers, with distinctions between younger caregivers of working age and those who are spouses demonstrating the need to individualise the provision of support for these caregivers.

### Study limitations

While there are a number of strengths to the study (it is the first survey that assessed the various aspects of the roles of caregivers and their quality of life in patients undergoing anti-VEGF therapy for nAMD; the sampling process captured a diverse range of caregivers that are likely to be representative of the caregiver population; and the use of an independent interviewer rather than a health care provider to reduce response bias), there are also some important limitations to consider.

It is important to recognise that the explanatory power of the study is limited to its design as a cross-sectional study. A study of caregiver burden either prospectively in relation to pre and post exposure to ranibizumab therapy or a comparison group of nAMD patients not receiving ranibizumab would have enhanced the explanatory power of the study. We were also not able to evaluate the input of other medical conditions on the total caregiver burden. However, we believe that our model has demonstrated that eye care related caregiver activities are an independent burden to caregivers. The study also did not use validated questionnaires that are specific for caregivers of patients with eye conditions. However, the items of the CRA have been formulated in such a way that they can be presented to informal caregivers of patients with chronic diseases. The comparison of CRA scores with those of other chronic conditions also indicate that the results obtained in this study is a true reflection of caregiver burden for nAMD requiring regular monthly hospital appointments.

There may be some centre bias to the study, as the participating sites have a high level of access to ranibizumab therapy for nAMD with relatively good visual outcomes compared to other centres around the world.[24–26] The models of care provided in other centres may be different. However, as these are generally considered to be centres of good practice it is likely that the level of caregiver support will be equivalent or poorer in other centres.

A final area of limitation could be a selection bias. The sample was one of convenience with ambulant clients who had volunteered. It is possible that volunteers with nAMD and their caregivers who were not overly burdened by their condition could have been more likely to volunteer, or the responses of caregivers may have been in a more positive direction than for the broader community of caregivers of people with nAMD. However, the effect of this would be to underplay (rather than overplay) the level of caregiver burden and the sampling methods are similar to most other studies that have examined quality of life in caregivers throughout the world.

In summary, this study provides an insight into the significant caregiver burden of patients receiving ranibizumab therapy for nAMD and the factors that contribute to the burden. It is important that services providing this therapy develop better information and supportive services for patients, together with an assessment of their needs. Future research in this area needs to validate these findings in different health systems in prospective comparative studies.
